# Gut microbiota modulation and immunity enhancement by *Bacillus amyloliquefaciens* NL1.2: A fiber-degrading probiotic isolated from native Thai swine

**DOI:** 10.14202/vetworld.2025.1487-1507

**Published:** 2025-06-10

**Authors:** Kittiya Khongkool, Malai Taweechotipatr, Sunchai Payungporn, Vorthon Sawaswong, Monthon Lertworapreecha

**Affiliations:** 1 Biotechnology Program, Faculty of Science and Digital Innovation, Thaksin University, Phatthalung Province 93210, Thailand; 2 Center of Excellence in Probiotic Research, Faculty of Medicine, Srinakharinwirot University, Wattana, Bangkok 10110, Thailand; 3 Center of Excellence in Systems Microbiology, Faculty of Medicine, Chulalongkorn University, Pathumwan, Bangkok 10330, Thailand; 4 Department of Biochemistry, Faculty of Science, Mahidol University, Bangkok 10400, Thailand; 5 Microbial Technology for Agriculture, Food, and Environment Research Center, Faculty of Science and Digital Innovation, Thaksin University, Phatthalung Province 93210, Thailand

**Keywords:** *Bacillus amyloliquefaciens*, fiber-degrading enzymes, gut microbiome modulation, mucosal immunity, native swine feces, probiotics

## Abstract

**Background and Aim:**

The pursuit of sustainable alternatives to antibiotic growth promoters has intensified interest in spore-forming probiotics with fiber-degrading capabilities. This study aimed to isolate, characterize, and evaluate the safety and functional properties of *Bacillus* spp. from native Thai swine, focusing on strains with probiotic potential and enzymatic activity for application in livestock nutrition.

**Materials and Methods:**

Spore-forming *Bacillus* isolates were obtained from fecal samples of backyard-raised native pigs. Isolates were screened for acid and bile tolerance, autoaggregation, hydrophobicity, biofilm formation, adhesion to Caco-2 cells, antimicrobial activity, and co-aggregation with pathogens. Enzyme production (cellulase, xylanase, and pectinase), hemolytic activity, and antibiotic susceptibility were also assessed. The most promising strain, *Bacillus amyloliquefaciens* NL1.2, was subjected to *in vivo* safety and efficacy evaluations in a mouse model, including assessments of toxicity, histopathology, secretory immunoglobulin A (IgA) levels, and gut microbiome modulation through full-length 16S ribosomal RNA sequencing.

**Results:**

*B. amyloliquefaciens* NL1.2 exhibited robust probiotic traits including high acid (115.05%) and bile (75.16%) tolerance, strong autoaggregation (65.99%), moderate hydrophobicity (34.13%), and effective adhesion (2.0%) to intestinal epithelial cells. It produced fiber-degrading enzymes (cellulase: 0.015 U/mL; xylanase: 0.522 U/mL; and pectinase: 0.374 U/mL) showed antimicrobial activity against *Enterohemorrhagic Escherichia coli*, *Enteropathogenic E. coli*, and *Salmonella* Typhimurium, and was non-hemolytic and antibiotic-sensitive. *In vivo*, NL1.2 induced no adverse effects and significantly elevated intestinal secretory IgA levels (p < 0.05). Microbiome analysis revealed enrichment of beneficial taxa (e.g., *Bacteroidetes* and *Barnesiella*) and reduction of potentially pathogenic taxa (e.g., *Helicobacter* and *Deferribacteres*).

**Conclusion:**

*B. amyloliquefaciens* NL1.2 is a safe, multifunctional probiotic with fiber-degrading, immunomodulatory, and gut microbiota-modulating properties. Its origin from native swine and broad functional attributes highlights its potential as a next-generation feed additive for sustainable animal production.

## INTRODUCTION

Feed accounts for a substantial proportion of operational costs in livestock production systems. To reduce these expenses, producers increasingly utilize industrial and agricultural by-products as alternative feed ingredients [1]. However, the structural complexity of these materials and the absence of sufficient endogenous digestive enzymes in animals often result in poor nutrient bioavailability and suboptimal feed efficiency. To address these limitations, exogenous enzymes are routinely incorporated into animal diets to improve nutrient digestibility, enhance growth performance, and promote gastrointestinal health by minimizing antinutritional factors and suppressing enteric pathogens. These enzymes are employed either individually or in synergistic combinations, depending on the species and physiological stage of the animals [2, 3].

Although subtherapeutic antibiotics have traditionally been used as growth promoters, their extensive use has raised serious concerns due to the emergence of antimicrobial resistance, disruption of commensal gut microbiota, and intestinal dysbiosis. This has prompted the search for safer and more sustainable alternatives. Probiotics, or direct-fed microbials, have gained considerable attention as promising agents to counteract these issues [4]. Defined as live microorganisms that confer health benefits to the host when administered in adequate quantities [5], probiotics offer multiple advantages. These include the inhibition of pathogen colonization, secretion of antimicrobial substances, enhancement of intestinal barrier integrity, modulation of host immune responses [6], and production of digestive enzymes [7], all of which contribute to improved growth performance and reduced incidence of gastrointestinal disorders.

Historically, probiotic research has focused predominantly on lactic acid bacteria, which, despite their efficacy, are strict anaerobes and exhibit limited stability during processing and storage due to their sensitivity to oxygen, heat, and low pH environments [8]. In contrast, spore-forming *Bacillus* species are now being recognized as robust probiotic candidates due to their enhanced viability, environmental resilience, and functional versatility [9, 10]. These Gram-positive, rod-shaped, endospore-forming bacteria thrive under both aerobic and anaerobic conditions [11], and are widely distributed in soil [12], aquatic environments [13], the human microbiome [14], animal gastrointestinal tracts [15, 16], and diverse food matrices [17, 18]. *Bacillus* species are known for synthesizing a variety of bioactive compounds - including digestive enzymes [19], antimicrobial peptides [20], and vitamins [21] - making them highly valuable in industrial and agricultural applications. Notably, *Bacillus* spores demonstrate superior resistance to gastrointestinal stress and maintain viability during feed processing and long-term storage [22], underscoring their suitability for use as next-generation probiotics in animal health.

Despite growing interest in probiotics as alternatives to antibiotic growth promoters in livestock, current research has disproportionately focused on lactic acid bacteria, which are limited by their sensitivity to environmental stressors and poor survivability during feed processing and gastrointestinal transit. Although *Bacillus* species have recently gained attention due to their spore-forming capabilities and robust functional profiles, the identification of strains that simultaneously possess strong probiotic properties, fiber-degrading enzymatic activity, and immunomodulatory potential remains insufficiently explored. In particular, few studies have systematically isolated and characterized native *Bacillu*s strains from non-industrial swine environments, which may serve as reservoirs for functionally superior probiotics due to their natural adaptation to high-fiber diets and minimal antibiotic exposure. Furthermore, limited data exist on the safety, mucosal immune responses, and gut microbiota modulation induced by such strains in *in viv*o systems. These knowledge gaps hinder the development of multifunctional, host-adapted probiotics capable of enhancing feed efficiency, gut health, and immune function in livestock.

This study aimed to isolate, characterize, and evaluate the safety and functional efficacy of spore-forming *Bacillu*s strains from the feces of native Thai pigs raised under backyard conditions. The objective was to identify candidate strains that exhibit key probiotic traits – such as acid and bile tolerance, adhesion capacity, and antimicrobial activity – alongside the production of fiber-degrading enzymes including cellulase, xylanase, and pectinase. The most promising strain, *Bacillus amyloliquefacien*s NL1.2, was further evaluated in a murine model to assess its *in viv*o safety, capacity to enhance mucosal immunity through secretory immunoglobulin A (IgA) production, and its modulatory effects on the gut microbiome through full-length 16S ribosomal RNA *(16S rRN*A) gene sequencing. The overarching goal was to establish the potential of *B. amyloliquefacien*s NL1.2 as a multifunctional probiotic feed additive for improving intestinal health, nutrient digestibility, and overall animal performance in sustainable livestock production systems.

## MATERIALS AND METHODS

### Ethical approval

All animal experiments were approved by the Animal Ethics Committee of Srinakharinwirot University (Approval No. COA/AE-003–2566) and were conducted in accordance with the institutional guidelines for the care and use of laboratory animals, and complied with the ARRIVE (Animal Research: Reporting of In Vivo Experiments) guidelines.

### Study period and location

The study was conducted from August 2022 to December 2023 for sample collection and *in vitro* assessment, followed by animal experiments and gut microbiome analysis from January 2024 to December 2024. A total of 24 fecal samples were collected from healthy native pigs raised in backyard farming systems across Krabi and Nakhon Si Thammarat provinces, southern Thailand. Isolation, phenotypic characterization, and *in vitro* assays – including functional property evaluation, enzyme activity screening, and safety assessments – were conducted at the Faculty of Science and Digital Innovation, Thaksin University, Phatthalung. *In vivo* evaluations using a murine model were performed at the Faculty of Medicine and the Center of Excellence in Probiotic Research, Srinakharinwirot University, Bangkok. Histopathological assessments were conducted at the Department of Pathology, Faculty of Medicine, Chulalongkorn University. Gut microbiome analyses were performed at the Center of Excellence in Systems Microbiology, Chulalongkorn University, Bangkok, Thailand.

### Sample collection and isolation of spore-forming bacteria

#### Sample collection

Fecal samples were obtained from Thai native pigs maintained under extensive backyard management in Krabi and Nakhon Si Thammarat. To minimize environmental contamination, pig pens were cleaned before sampling. Fresh feces were collected in the early morning immediately after defecation using sterile spatulas, avoiding ground contact. Each sample was aseptically transferred to a sterile screw-cap tube, placed in an icebox, and transported to the laboratory within 12–24 h. All samples were processed immediately under aseptic conditions to maintain microbial viability.

#### Isolation of spore-forming bacteria

Bacterial isolation was performed as described in previous studies by Barbosa *et al*. [23] and Singh *et al*. [24]. One gram of each fecal sample was homogenized in 9 mL of sterile 0.85% (w/v) normal saline by orbital shaking at 180 rpm for 1 h at 37°C. To select spore-formers, the homogenate was subjected to heat treatment at 65°C for 30 min to eliminate vegetative cells. The treated suspension was serially diluted tenfold in saline, and 100 μL of appropriate dilutions were spread on Luria-Bertani (LB) agar (HiMedia, India) and incubated at 37°C for 24 h. Colonies with distinct morphology were subcultured repeatedly to obtain pure isolates. Preliminary screening involved Gram staining, colony morphology, and spore formation. Isolates identified as Gram-positive, rod-shaped, and spore-forming were stored in LB broth with 20% (v/v) glycerol at −80°C for long-term preservation.

### Species exclusion and molecular identification

#### DNA extraction

Bacterial genomic DNA was extracted from overnight LB broth cultures using the GF-1 Bacterial DNA Extraction Kit (Vivantis Technologies, Malaysia; Cat. No. GF-BA-100). Cultures were centrifuged at 9,860 × *g* for 5 min at 4°C, and DNA purity and concentration were assessed using a NanoDrop Lite Spectrophotometer (Thermo Scientific, USA). DNA samples were diluted to the working concentration and stored at −20°C.

#### Exclusion of the Bacillus cereus group

To ensure biosafety, isolates were screened for *B. cereus* group members (*B. cereus*, *Bacillus* anthracis, and *Bacillus* *thuringiensis*) through polymerase chain reaction (PCR) targeting the *motB* gene, using primers BCFomp1 (5’–ATCGCCTCGTTGGATGACGA–3’) and BCRomp1 (5’–CTGCATATCCTACCGCAGCTA–3’), as previously described by Oliwa-Stasiak *et al*. [25]. PCR was performed under the following conditions: Initial denaturation at 94°C for 5 min; 30 cycles of 94°C for 30 s, 60.5°C for 45 s, and 72°C for 1 min; with a final extension at 72°C for 7 min. Amplicons were resolved on a 1.5% (w/v) agarose gel (0.5× Tris-Borate-EDTA buffer and visualized at 100 V for 30 min alongside a 1-kb DNA ladder. *B. cereus* American Type Culture Collection (ATCC) 14579 served as the positive control. Isolates testing positive were excluded from further analysis.

#### 16S rRNA gene sequencing

Isolates that passed exclusion screening underwent species identification through *16S rRNA* gene sequencing using universal primers 27F (5’–AGAGTTTGATCCTGGCTCAG–3’) and 1492R (5’–GGTTACCTTGTTACGACTT–3’) [26]. PCR conditions were as follows: 95°C for 3 min; 30 cycles of 95°C for 1 min, 55°C for 1 min, and 72°C for 1 min; followed by a final extension at 72°C for 5 min. PCR products (~1,466 bp) were resolved by agarose gel electrophoresis and purified using the GF-1 AmbiClean Kit (Vivantis Technologies; Cat. no. GF-GC-100). Sequencing was performed by 1^st^ BASE DNA Sequencing Services (BaseAsia, Singapore). Species identity was determined through-Basic Local Alignment Search Tool (BLAST, https://blast.ncbi.nlm.nih.gov/Blast.cgi) alignment against the-National Center for Biotechnology Information database (https://www.ncbi.nlm.nih.gov), using a similarity threshold of ≥98%. Sequences were submitted to GenBank, and accession numbers were assigned. The identified probiotic strain was deposited at the Thailand Bioresource Research Center.

### In vitro probiotic characterization

#### Acid tolerance assay

The acid tolerance of vegetative cells was assessed using a modified protocol based on previously described methods by Hmani *et al*. [27]. Overnight cultures of the bacterial isolates were grown in LB broth at 37°C and harvested by centrifugation at 5,000 × *g* for 10 min at 4°C. The resulting cell pellets were washed and resuspended in sterile normal saline solution. Cell turbidity was adjusted to the 0.5 McFarland standard using a Den-1B suspension turbidity detector (BioSan, Riga, Latvia). Subsequently, 1 mL of the standardized cell suspension was inoculated into 9 mL of LB broth acidified to pH 3 using 1 M HCl and incubated at 37°C for 3 h. Viable cell counts were determined by plating ten-fold serial dilutions on LB agar and incubating the plates at 37°C for 18–24 h. All assays were performed in technical triplicate. *Bacillus subtilis* KMP, a commercial probiotic strain, was used as the reference control (strain-specific details withheld for proprietary reasons). Acid tolerance was expressed as the percentage of viable cells remaining after 3 h relative to the baseline (0-h) count. Isolates with survival rates >50% were classified as acid-tolerant and selected for further evaluation.

#### Bile salt tolerance assay

Bile tolerance was determined following a protocol adapted from a previously validated method by Sirichokchatchawan *et al*. [28], with minor modifications to the incubation duration. Bacterial cultures were prepared as described in the acid tolerance assay. A 1 mL aliquot of each standardized suspension was inoculated into 9 mL of LB broth supplemented with 1% (w/v) bile (HiMedia) and incubated at 37°C. Viable counts were measured immediately (0 h) and after 3 h by the spread plate method. Each test was conducted in technical triplicate. *B. subtilis* KMP served as the reference strain. Bile tolerance was assessed by comparing the colony-forming units (CFU) at 3 h with the initial counts.

#### Auto-aggregation assay

The auto-aggregation ability of the isolates was evaluated using a modified version of a previously established protocol by Jeon *et al*. [29]. Active bacterial cultures were harvested, washed with phosphate-buffered saline (PBS), and resuspended to an optical density (OD600) of 0.8 ± 0.2. The suspensions were incubated statically at 37°C. At 2, 4, 8, and 24-h time points, 200 μL of the upper phase was carefully aspirated and measured at 600 nm using a microplate reader (Biochrom Asys UVM 340, England). Auto-aggregation was calculated using the formula:

Auto-aggregation (%) = [1 – (A_t_ ÷ A_0_)] × 100

Where A_t_ is the absorbance at each time point and A_0_ is the initial absorbance. All assays were performed in triplicate, with *B. subtilis* KMP used as the control. Auto-aggregation capacity was categorized as low (16%–35%), intermediate (35%–50%), or high (>50%) based on established thresholds [30].

### Cell surface hydrophobicity assay

The cell surface hydrophobicity of bacterial isolates was assessed using the microbial adhesion to hydrocarbon (MATH) method, as previously described by Fonseca *et al*. [31], with xylene serving as the hydrophobic phase. Overnight LB broth cultures of each isolate were harvested by centrifugation, washed twice with sterile PBS, pH 7.2, and resuspended in PBS. The OD of the suspension was adjusted and recorded at 600 nm (*A<sub>before</sub>*) using a microplate reader.

Subsequently, 1 mL of xylene was added to 3 mL of the bacterial suspension, and the mixture was vortexed vigorously for 2 min to allow interaction between the bacterial cells and the hydrocarbon phase. The mixture was then incubated at 37°C for 1 h to permit phase separation. After incubation, the aqueous phase was carefully collected, and its OD at 600 nm (*A<sub>after</sub>*) was recorded.

Cell surface hydrophobicity (%) was calculated using the following formula:

Cell surface hydrophobicity (%) = [1 – (A_after_ ÷ A_before_)] × 100

All measurements were performed in technical triplicates. *B. subtilis* KMP was used as the reference control strain. Based on the percentage values, hydrophobicity was classified as strong (>50%), moderate (20%–50%), or weak (<20%) adhesion to hydrocarbons, following standard interpretive guidelines [32].

### Biofilm formation assay

Biofilm-forming ability was assessed using the standard 96-well microtiter plate assay, following a previously established protocol by Coffey and Anderson [33]. Bacterial isolates were cultured in LB broth and incubated overnight at 37°C with shaking at 180 rpm in a shaking incubator. Cells were harvested, washed, and adjusted to a 0.5 McFarland standard (~1 × 10^8^ CFU/mL). The suspension was diluted 1:100 in LB broth supplemented with 5% (w/v) glucose. A 100 μL aliquot was dispensed into each well of a flat-bottom 96-well polystyrene plate and incubated at 37°C for 72 h under static conditions.

Following incubation, wells were gently washed twice with sterile distilled water and air-dried at 25°C. Biofilms were stained with 0.1% (w/v) crystal violet for 10 min, rinsed with water to remove excess stain, and dried again. The bound dye was solubilized with 95% ethanol, and the absorbance was measured at 570 nm using a microplate reader. Each assay was performed in technical triplicate. *B. subtilis* KMP was used as a reference control strain.

### Adhesion to caco-2 cells

Caco-2 cells (ATCC Human Tumor Bank-37) were cultured in Dulbecco’s Modified Eagle’s Medium (DMEM) (DMEM; Invitrogen, USA) supplemented with 10% (v/v) heat-inactivated fetal bovine serum, 100 U/mL penicillin, and 100 μg/mL streptomycin. Cells were maintained at 37°C in a humidified atmosphere with 5% CO_2_ and subcultured on reaching 80%–90% confluency.

For adhesion assays, Caco-2 cells were seeded in 24-well plates at 2 × 10^5^ cells/mL and cultured for 21 days to allow differentiation. One hour before the assay, the medium was replaced with serum-free DMEM. Overnight bacterial cultures were harvested, washed in PBS, and resuspended in DMEM to 1 × 10^8^ CFU/mL. After washing the monolayers twice with PBS, 1 mL of bacterial suspension was added to each well and incubated for 2 h at 37°C with 5% CO_2_.

Post-incubation, non-adherent bacteria were removed by washing twice with PBS. Cells were lysed with 1 mL of 0.1% Triton X-100 in PBS and incubated for 10 min. The lysates were serially diluted and plated on LB agar to determine the number of adherent bacteria. Adhesion percentage was calculated relative to the initial inoculum. All assays were conducted in technical triplicate.

### Anti-pathogenic activity assay

The antimicrobial activity of *Bacillus* isolates was evaluated using the agar well diffusion method as described previously by Lertcanawanichakul and Sawangnop [34]. Pathogenic indicator strains included *Enterohemorrhagic Escherichia coli* (EHEC) SC2451–1, Enteropathogenic *E. coli* (EPEC) SC2451–2, and *Salmonella enterica* serovar Typhimurium SC2451–3, including an ESBL-producing strain [35].

Each isolate and pathogen was cultured separately in LB broth at 37°C for 24 h. Cell-free culture supernatants (CFCSs) were obtained by centrifugation at 5,000 × *g* for 10 min at 4°C. Petri dishes containing 25 mL of sterile LB agar were inoculated with 100 μL of pathogen suspension adjusted to 0.5 McFarland standard. Wells (6 mm diameter) were punched into the agar and filled with 80 μL of either native or pH-neutralized CFCS. LB broth served as the negative control.

Plates were incubated at 37°C for 16–18 h, after which the diameter of the inhibition zones was measured. Each assay was performed in triplicate to ensure reproducibility.

### Co-aggregation assay

Co-aggregation between *Bacillus* isolates and pathogenic strains (EHEC SC2451–1, EPEC SC2451–2, and *Salmonella* Typhimurium SC2451–3) was evaluated following a modified version of a previously published method by Sirichokchatchawan *et al*. [28]. Overnight cultures of each isolate and pathogen were harvested, washed, and resuspended in PBS to an OD_600_ of 0.8 ± 0.2.

Equal volumes (2 mL each) of *Bacillus* and pathogen suspensions were mixed, vortexed briefly, and incubated at 37°C without agitation for 4 h. Post-incubation, 200 μL of the upper suspension was carefully removed, and OD_600_ was recorded. The degree of co-aggregation was calculated using the formula:


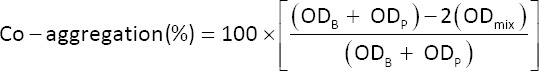


Where OD_B_ and OD_P_ are absorbances in control tubes containing only *Bacillus* species or the indicator pathogenic strain, respectively, and OD_mix_ is the absorbance of the mixed suspension at 4 h. The assay was performed in triplicate.

### Hemolytic activity

Hemolytic activity was assessed by streaking each bacterial isolate onto ready-to-use sheep blood agar plates (M and P IMPEX, Bangkok, Thailand) and incubating them at 37°C for 24 h. Following incubation, plates were examined for the type of hemolysis exhibited. Clear zones surrounding colonies were indicative of β-hemolysis, greenish zones of α-hemolysis, and the absence of any discoloration or clearing around colonies denoted γ-hemolysis (non-hemolytic) [36]. Only non-hemolytic (γ-hemolysis) strains were considered safe for further probiotic evaluation.

### Antibiotic susceptibility test

Antibiotic susceptibility was determined using the Kirby-Bauer disc diffusion method according to Clinical and Laboratory Standards Institute (CLSI) guidelines [37]. Ten antibiotics (HiMedia) were tested: Ampicillin (25 μg), cephalothin (30 μg), chloramphenicol (30 μg), ciprofloxacin (10 μg), erythromycin (15 μg), gentamicin (120 μg), norfloxacin (10 μg), streptomycin (10 μg), tetracycline (10 μg), and vancomycin (30 μg).

Each isolate was adjusted to 0.5 McFarland turbidity and uniformly spread onto Mueller–Hinton agar (HiMedia) plates. Antibiotic discs were placed on the inoculated surface, and plates were incubated at 35 ± 1°C for 18 ± 2 h. The diameter of the inhibition zones (including the 6 mm disc) was measured in millimeters and interpreted as susceptible (S), intermediate (I), or resistant (R) per CLSI breakpoints. Each assay was conducted in technical triplicate.

### Enzyme screening and activity measurement

#### Qualitative screening of fiber-degrading enzymes

All *Bacillus* isolates were screened for the production of cellulase, xylanase, and pectinase using agar-based assays. Bacterial cultures were grown overnight, washed, and adjusted to 0.5 McFarland standard in PBS.

- Cellulase activity was detected on carboxymethylcellulose (CMC) agar composed of 2.0 g/L NaNO_3_, 1.0 g/L K_2_HPO_4_, 0.5 g/L MgSO_4_, 0.5 g/L KCl, 2.0 g/L CMC (Sigma-Aldrich), 0.2 g/L peptone, and 20.0 g/L agar. After incubation at 28°C for 48 h, plates were flooded with 0.01 M iodine-potassium iodide (I_2_-KI) solution. Clear zones around colonies indicated cellulolytic activity [38].
- Xylanase activity was assessed on xylan agar containing 0.05 g/L MgSO_4_·7H_2_O, 0.05 g/L NaCl, 0.01 g/L CaCl_2_, 0.2 g/L yeast extract, 0.5 g/L peptone, 10.0 g/L birchwood xylan (Sigma-Aldrich), and 20.0 g/L agar. After 72 h at 30°C, plates were stained with 0.4% Congo red and destained with 1 M NaCl. Clear halos indicated xylanase activity [39].
- Pectinase activity was tested using pectin agar composed of 1.0 g/L NaNO_3_, 1.0 g/L KCl, 1.0 g/L K_2_HPO_4_, 0.5 g/L MgSO_4_, 0.5 g/L yeast extract, 10.0 g/L citrus pectin (Sigma-Aldrich), and 20.0 g/L agar, adjusted to pH 7.0. After 48 h of incubation at 37°C, plates were overlaid with 0.01 M I_2_-KI solution. Clear zones indicated pectinolytic activity [40].

### Quantitative enzyme activity assays

For enzyme quantification, isolates were inoculated into broth media formulated identically to the respective screening agars, diluted 1:10 from the 0.5 McFarland suspension, and incubated for 24 h. CFCS were collected by centrifugation and used as crude enzyme extracts.

Enzymatic activity was measured using the 3,5-dinitrosalicylic acid (DNS) method to quantify reducing sugars [41]. Specific substrates were used: Cellulose for cellulase, birchwood xylan for xylanase, and citrus pectin for pectinase. Each reaction mixture was incubated at:

- 50°C for 30 min (cellulase and xylanase assays),
- 55°C for 30 min (pectinase assay).

The reaction was terminated by adding DNS reagent and boiling for 5 min. After cooling, absorbance was measured at 540 nm. Glucose, xylose, and D-galacturonic acid (all from Sigma-Aldrich) served as standards.

Enzyme activity was expressed in units (U), defined as the amount of enzyme required to release 1 μmol of reducing sugar per minute under assay conditions. All measurements were performed in triplicate.

### *In vivo* safety assessment of mice

#### Animals, housing, and husbandry

Specific-pathogen-free male (Institute of Cancer Research: mice (*Mus musculus*), aged 7 weeks and weighing 30 ± 10 g, were procured from the National Laboratory Animal Center, Mahidol University (Bangkok, Thailand). Mice were acclimatized for 1 week before the experimental period. Animals were housed in conventional polycarbonate cages (three mice per cage) under controlled conditions: 24°C ± 1°C temperature, 55% ± 10% relative humidity, and a 12:12-h light-dark cycle. Bedding consisted of dried water hyacinth, changed every 3 days. Mice had *ad libitum* access to sediment-filtered drinking water and a standard commercial rodent diet (082G, NLAC-MU, Bangkok, Thailand).

#### Experimental design

After acclimatization, mice were randomly assigned to either the control or treatment group (n = 6 per group) using a computer-generated randomization sequence. The control group received 100 μL of sterile PBS (pH 7.2) orally, while the treatment group was administered 100 μL of *B. amyloliquefaciens* NL1.2 suspension (1 × 10^11^ CFU/mL) daily for 30 consecutive days. The selected dosage was informed by *in vitro* adhesion data and accounted for anticipated microbial loss during gastrointestinal transit, ensuring viable delivery of ≥1 × 10^9^ CFU/mL *in vivo*. Blinding was not implemented due to logistical constraints associated with daily dosing and observation.

#### Clinical observations

Throughout the 30-day study period, mice were observed daily for clinical signs of toxicity, including alterations in physical appearance (fur, mucosa, and eyes), behavior (activity and gait), autonomic responses (salivation and tremors), and gastrointestinal symptoms (e.g., diarrhea). Mortality, injuries, feed consumption (3–6 g/mouse/day), and body weight (20–40 g) were also monitored by trained personnel following standard protocols [42].

### Secretory IgA assay

At the end of the study, intestinal secretions were collected by flushing the small intestine (from the gastro-duodenal region to the ileocecal junction) with 2 mL of sterile PBS (pH 7.2). The collected fluids were centrifuged at 9,860 × *g* for 10 min at 4°C, and supernatants were filtered through 0.22 μm sterile syringe filters. Secretory IgA levels were measured using a Mouse IgA Uncoated ELISA Kit (Invitrogen, USA; Cat. no. 88-50450-88) following the manufacturer’s protocol. The standard curve ranged from 0.39 to 25.00 ng/mL, with a detection limit of 0.39 ng/mL.

Plates were coated with anti-mouse IgA monoclonal antibodies and incubated overnight at 4°C, followed by blocking and incubation with standards or samples for 2 h at 25°C. After washing, Horseradish Peroxidase (HRP)-conjugated polyclonal anti-IgA (Thermo Fisher Scientific, USA), was added and incubated for 1 h. 3,3′,5,5′-Tetramethylbenzidine substrate was added for color development, and reactions were stopped with 2 N H_2_SO_4_. Absorbance was read at 450 nm.

### Histopathological examination

Following overnight fasting, mice were anesthetized using isoflurane (Attane^™^, VetEquip, USA) and humanely euthanized. Internal organs (small intestine, colon, liver, and spleen) were dissected, rinsed in cold PBS, and fixed in 4% paraformaldehyde.

Tissues were processed using a Leica TP1020 tissue processor, embedded in paraffin with a MEDITE TES Valida system (Medite GmbH, Germany), and sectioned at 4–6 μm using a HistoCore MULTICUT (Leica Biosystems, Germany), rotary microtome. Sections were mounted on slides, deparaffinized, rehydrated, and stained with hematoxylin and eosin. Slides were examined under an Olympus UC50 light microscope (Olympus Corporation, Japan), by an independent pathologist blinded to the treatment groups.

### Gut microbiome analysis

#### Sample collection and DNA extraction

Fecal contents were collected from the colon and preserved in DNA/RNA Shield (Zymo Research, USA; Cat. No. R1100-250). DNA was extracted using the ZymoBIOMICS DNA Miniprep Kit, (Zymo Research Corporation, USA., Cat. No. D4300) as per the manufacturer’s protocol.

#### 16S rRNA gene sequencing

The full-length 16S rDNA sequencing was performed following a previously established method by Sawaswong *et al*. [43]. Briefly, 16S rDNA (V1–V9 regions) was amplified using primers 27F/1492R with nanopore adaptors Amplicons were barcoded using the PCR Barcoding Expansion 1–96 Kit (Oxford Nanopore Technologies; Cat. no. EXP-PBC096), purified with QIAquick PCR Kit (Qiagen; Cat. no. 28104), quantified with the-Quant-iT™ dsDNA HS Assay Kit (Thermo Fisher Scientific; Invitrogen™, USA; Cat. no. Q33120), and pooled to 5 μg. Libraries were purified using 0.5× AMPure XP beads and ligated using the Ligation Sequencing Kit (ONT; Cat. No. SQK-LSK112), then se- quenced on a MinION Mk1C with R10.4 (Q20+) flow cell.

#### Bioinformatics and statistical analysis

Raw FAST5 reads were base-called using Guppy v6.1.2 in super-accuracy mode. Demultiplexing and adapter trimming were performed with Porechop v0.2.4. Taxonomic clustering and identification were conducted using NanoCLUST through the Nextflow pipeline (Instituto Tecnológico y de Energías Renovables, Spain), referencing the Ribosome Database Project 16S database (v11.5, Michigan State University, USA) with a minimum cluster size of 20.

Data normalization was performed using total sum scaling through MicrobiomeAnalyst (https://www.microbiomeanalyst.ca). Alpha diversity (Shannon, Simpson, Chao1) and beta diversity (Bray-Curtis and Jaccard distances) were assessed. Statistical testing included the Shapiro-Wilk test, Mann-Whitney U-test, and Hutcheson t-test (for Shannon index). Principal Coordinates Analysis (PCoA) was used for visualization, and Permutational Multivariate Analysis of Variance (PERMANOVA) was applied to test group-wise differences. Differential taxa were identified using Linear discriminant analysis effect size (LEfSe) (Linear discriminant analysis score >2.0, p < 0.1), with false discovery rate correction through Benjamini-Hochberg adjustment.

### Statistical analysis

All *in vitro* assays were performed in triplicate, and results are expressed as mean ± standard deviation. *In vivo* data were analyzed using independent samples t-tests (Statistical Package for the Social Sciences [SPSS] Statistics v22, SPSS Inc., USA). p < 0.05 was considered statistically significant.

## RESULTS

### Isolation and identification of spore-forming *Bacillus* strains

From 24 fecal samples collected from native swine, a total of 123 presumptive *Bacillus* isolates were obtained. All isolates were Gram-positive, catalase-positive, and rod-shaped bacteria exhibiting endospore formation, typically arranged in chains.

PCR screening using *motB*-specific primers excluded 94 isolates as members of the *B. cereus* group. The remaining 29 isolates were further identified by *16S rRNA* gene sequencing and BLAST analysis, revealing the presence of several species, including *Bacillus megaterium, Bacillus licheniformis, Bacillus stratosphericus, Bacillus paramycoides, B. amyloliquefaciens, B. subtilis*, and *Bacillus velezensis*.

### Functional probiotic properties and *in vitro* safety profiles

#### Acid and bile tolerance

**Figure 1:**
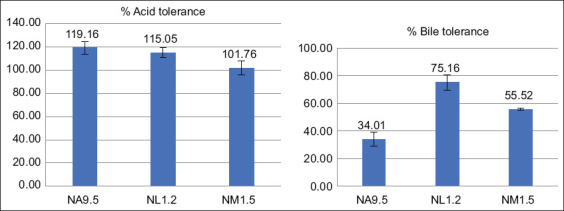
Acid and bile tolerance of *Bacillus* species in Thai native swine feces with survival rates exceeding 50%.

Three isolates demonstrated acid tolerance with survival rates exceeding 50% after 3 h at pH 3: *B. megaterium* NA9.5 (119.16% ± 5.63%), *B. amyloliquefaciens* NL1.2 (115.05% ± 4.06%), and *B. subtilis* NM1.5 (101.76% ± 6.09%). For bile tolerance, only NL1.2 (75.16% ± 5.62%) and NM1.5 (55.52% ± 0.74%) exhibited survival above the 50% threshold (Figure 1).

#### Auto-aggregation and cell surface hydrophobicity

Auto-aggregation increased progressively over 24 h, with NL1.2 and NM1.5 showing high values of 65.99% and 64.68%, respectively (Table 1). No statistically significant difference was observed (p > 0.05). NL1.2 showed moderate hydrophobicity (34.13%), while NM1.5 exhibited weak hydrophobicity (17.29%) (Table 1).

**Table 1:** Adhesion ability of *Bacillus amyloliquefaciens* NL1.2 and *Bacillus subtilis* NM1.5.

Strains	Autoaggregation ability (%)				Cell-surface hydrophobicity (%)	Adhesion of Caco-2 cells (%)	Biofilm production

	2 h	4 h	8 h	24 h			
NL1.2	24.78 ± 0.99^a^	29.60 ± 1.64^a^	59.75 ± 1.39^a^	65.99 ± 0.47^a^	34.13 ± 0.32^a^	2.0 ± 0.14^a^	0.76 ± 0.02^b^
NM1.5	16.78 ± 1.09^b^	27.81 ± 0.11^a^	56.61 ± 1.47^b^	64.68 ± 1.67^a^	17.29 ± 1.50^b^	0.6 ± 0.01^b^	1.39 ± 0.03^a^

Values are mean ± SD of three independent determinations (n = 3) of each sample. Different superscript letters in the same column indicate the significant difference (p < 0.05). SD=Standard deviation

#### Biofilm formation and adhesion to caco-2 cells

Both strains formed biofilms; however, NM1.5 demonstrated significantly higher biofilm formation (OD_570_ = 1.39) than NL1.2 (OD_570_ = 0.76; p < 0.05) (Table 1). Adhesion rates to Caco-2 cells were low overall, though NL1.2 adhered significantly better (2.0%) than NM1.5 (0.6%; p < 0.05) (Table 1).

#### Antagonistic activity and co-aggregation

Only NL1.2 exhibited anti-pathogenic activity against EHEC, EPEC, and *Salmonella* Typhimurium (Table 2). Co-aggregation assays showed both strains exhibited moderate co-aggregation (32.84%–40.96%). Strain-specific differences were noted, particularly with EPEC and *Salmonella* Typhimurium (p < 0.05) (T*abl*e 2).

**Table 2:** Anti-pathogenic activity of *Bacillus amyloliquefaciens* NL1.2 and *Bacillus subtilis* NM1.5.

Strains	Anti-pathogenic activity (zone of inhibition; mm)			Co-aggregation with indicator pathogens (%)		
	
	EHEC	EPEC	ST	EHEC	EPEC	ST
NL1.2	14.33 ± 0.57	12.33 ± 0.57	9.0 ± 0.00	40.96 ± 1.38^a^	34.52 ± 1.64^b^	34.76 ± 0.53^a^
NM1.5	ND	ND	ND	39.85 ± 0.28^a^	37.87 ± 0.22^a^	32.84 ± 0.70^b^

Values are mean ± SD of three independent determinations (n = 3) of each sample. ST=*Salmonella* Typhimurium SC2451-3, ND=Not detectable. Different superscript letters in the same column indicate the significant difference (p < 0.05). SD=Standard deviation, EHEC=*Enterohemorrhagic Escherichia coli*, EPEC=*Enteropathogenic Escherichia coli*

#### Hemolytic activity and antibiotic susceptibility

Both strains were non-hemolytic (γ-hemolysis). NL1.2 was susceptible to all tested antibiotics, while NM1.5 exhibited resistance to erythromycin (Table 3).

**Table 3:** Safety profiles of *Bacillus amyloliquefaciens* NL1.2 and *Bacillus subtilis* NM1.5.

Strains	Susceptibility to antibiotics										Hemolytic activity

	NX	C	CIP	CEP	TE	HLG	AMP	E	S	VA	
NL1.2	24 (S)	30 (S)	37 (S)	40 (S)	30 (S)	31 (S)	34 (S)	30 (S)	23 (S)	22 (S)	γ-hemolytic
NM1.5	29 (S)	27 (S)	36 (S)	23 (S)	32 (S)	31 (S)	22 (S)	6 (R)	17 (S)	22 (S)	γ-hemolytic

NX=Norfloxacin, CIP=Ciprofloxacin, HLG=Gentamicin, E=Erythromycin, TE=Tetracycline, C=Chloramphenicol, CEP=Cephalothin, AMP=Ampicillin, S=Streptomycin, VA=Vancomycin. Susceptibility (diameter of clear zone): S=Sensitive, I=Intermediate, R=Resistance

### Production of fiber-degrading enzymes

Both strains produced cellulase (0.015 U/mL). However, NM1.5 exhibited significantly higher xylanase (0.833 U/mL) and pectinase (0.668 U/mL) activity than NL1.2 (0.522 and 0.374 U/mL, respectively; p < 0.05) (Table 4).

**Table 4:** Fiber-degrading enzyme production by *Bacillus amyloliquefaciens* NL1.2 and *Bacillus subtilis* NM1.5.

Strains	Enzyme activity (U/mL)		

	Cellulase	Xylanase	Pectinase
NL1.2	0.015 ± 0.00^a^	0.522 ± 0.01^b^	0.374 ± 0.05^b^
NM1.5	0.015 ± 0.00^a^	0.833 ± 0.01^a^	0.668 ± 0.04^a^

Values are mean ± SD of three independent determinations (n = 3) of each sample. Different superscript letters in the same column indicate the significant difference (p < 0.05). SD=Standard deviation

### *In vivo* safety and immunomodulatory evaluation

#### Clinical observations

Oral administration of NL1.2 (1 × 10^11^ CFU/mL/day) for 30 days elicited no signs of toxicity, behavioral changes, weight loss, or gastrointestinal symptoms. Body weights and feed intake remained within normal physiological ranges (Table 5).

**Table 5:** General observations from an oral toxicity study of *Bacillus amyloliquefaciens* NL1.2 in mice.

Treatment	Body weight (g)			

	Week 1	Week 2	Week 3	Week 4
Control	35.41 ± 0.89^a^	36.68 ± 1.93^a^	37.12 ± 2.07^a^	37.86 ± 1.65^a^
NL1.2	34.64 ± 0.78^a^	35.36 ± 0.66^a^	36.32 ± 0.68^a^	36.85 ± 0.38^a^

**Treatment**	**Feed consumption (g/mouse/day)**			

	**Week 1**	**Week 2**	**Week 3**	**Week 4**

Control	4.51 ± 0.26^a^	3.96 ± 0.28^a^	3.96 ± 0.24^a^	3.84 ± 0.36^a^
NL1.2	4.45 ± 0.14^a^	3.93 ± 0.29^a^	3.94 ± 0.12^a^	3.80 ± 0.17^a^

**Treatment**	**Mortality (%)**			

	**Week 1**	**Week 2**	**Week 3**	**Week 4**

Control	ND	ND	ND	ND
NL1.2	ND	ND	ND	ND

**Treatment**	**General observations**			

	**Week 1**	**Week 2**	**Week 3**	**Week 4**

Control	Normal	Normal	Normal	Normal
NL1.2	Normal	Normal	Normal	Normal

Values are mean ± SD of three independent determinations (n = 6) of each sample. ND=Not detectable. Different superscript letters in the same column indicate the significant difference (p < 0.05). SD=Standard deviation

#### Histopathological findings

**Figure 2:**
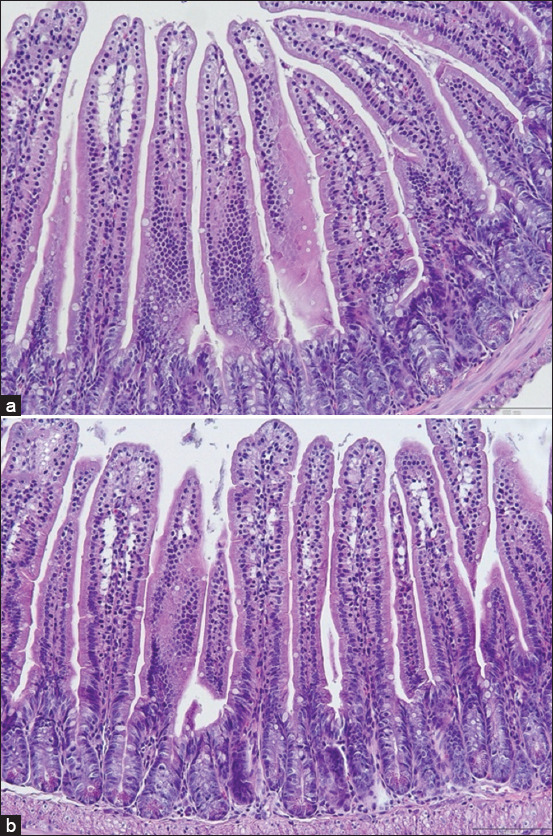
Histological examination of the small intestine of mice: (a) control group and (b) *Bacillus amyloliquefaciens* NL1.2 treatment group. Sections were observed at a magnification of 20×.

**Figure 3:**
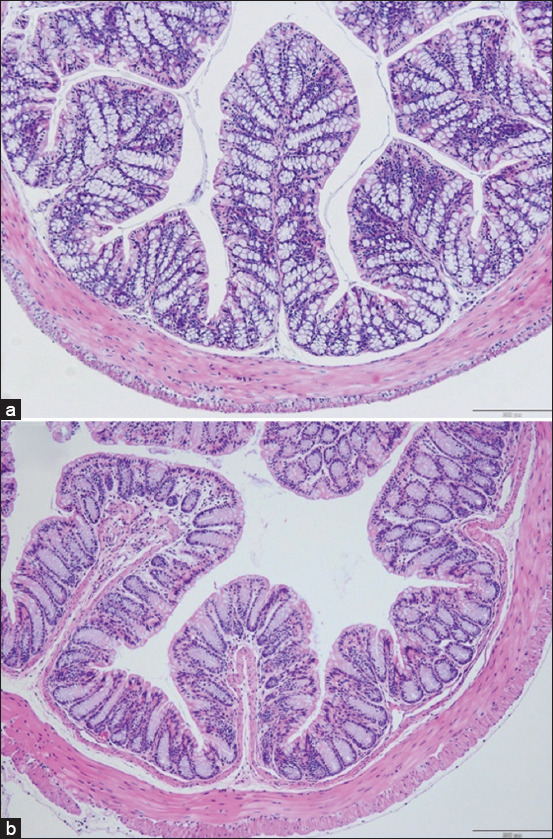
Histological examination of the colon of mice: (a) control group and (b) *Bacillus amyloliquefaciens* NL1.2 treatment group. Sections were viewed at 10× magnification.

**Figure 4:**
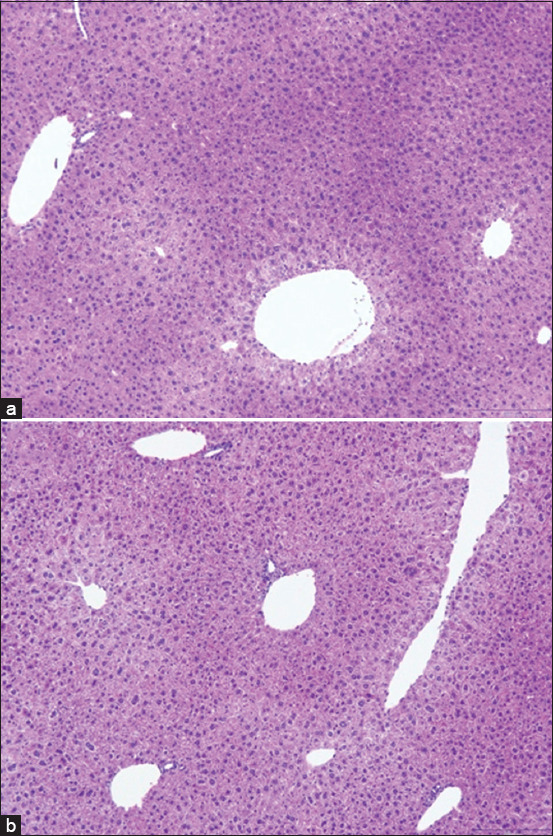
Histological examinations of the liver in mice: (a) control group and (b) *Bacillus amyloliquefaciens* NL1.2 treatment group. Sections were viewed at 10× magnification.

**Figure 5:**
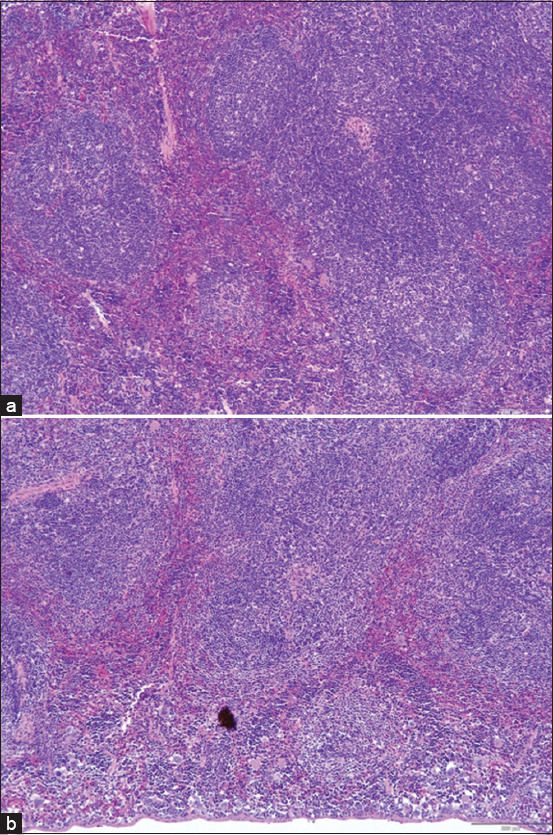
Histological examinations of the spleens of mice in the (a) control group and (b) *Bacillus amyloliquefaciens* NL1.2 treatment group. Sections were viewed at 10× magnification.

No histological abnormalities (e.g., necrosis, inflammation, and ulceration) were observed in the intestine, colon, liver, or spleen of NL1.2-treated mice, indicating no bacterial translocation or systemic toxicity (Figures 2–5).

#### Secretory IgA response

**Figure 6:**
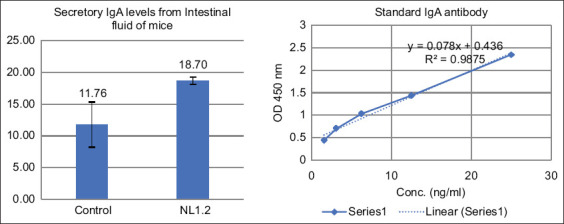
Total secretory immunoglobulin A levels in mice of the control and *Bacillus amyloliquefaciens* NL1.2 groups. Asterisks (*) indicate statistically significant differences (p < 0.05).

NL1.2 treatment significantly increased intestinal secretory IgA levels (18.70 ± 0.57 ng/mL) compared to the control group (11.76 ± 3.56 ng/mL; p < 0.05), suggesting enhanced mucosal immunity (Figure 6).

### Effects on gut microbiota composition

#### Taxonomic distribution

**Figure 7:**
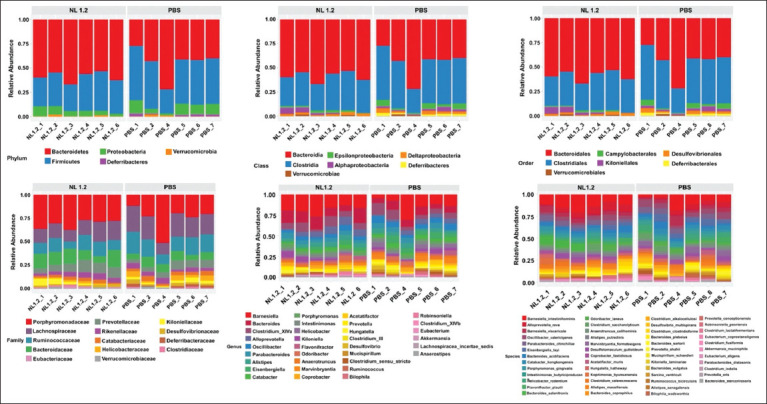
The stacked bar charts illustrate the relative abundance profiles across all taxonomic levels for mice in the *Bacillus amyloliquefaciens* NL1.2 group compared with the control (phosphate-buffered saline) group.

NL1.2 administration altered gut microbiome composition at multiple taxonomic levels (Figure 7). At the phylum level, NL1.2-treated mice had a higher abundance of *Bacteroidetes* (59.97%) and reduced *Firmicutes* (33.77%) and *Proteobacteria* (5.93%) compared to controls. The control group displayed increased *Deferribacteres* (1.80%).

At genus and species levels, NL1.2 enhanced the relative abundance of *Barnesiella intestinihominis*, *Bacteroides salanitronis*, and *Alloprevotella rava*. Potentially harmful genera such as *Mucispirillum*, *Helicobacter*, and *Eisenbergiella* were reduced or absent.

#### LEfSe analysis

**Figure 8:**
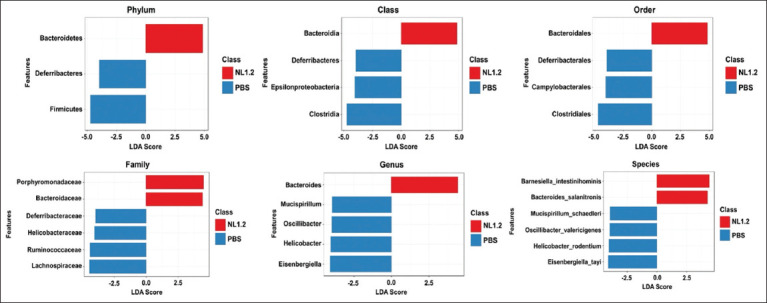
Linear discriminant analysis (LDA) effect size (LEfSe) comparison of differentially abundant bacterial taxa between *Bacillus amyloliquefaciens* NL1.2-treated mice and the control group. Horizontal bars show effect sizes, with blue indicating control-enriched taxa and red indicating *Bacillus*-enriched taxa. The bar length reflects the LDA score.

LEfSe revealed significantly enriched taxa in the NL1.2 group, including *Bacteroidetes, Bacteroidales, Bacteroidaceae, Porphyromonadaceae*, and *B. intestinihominis*. Conversely, the control group had higher levels of Deferribacteres, Clostridia, Helicobacteraceae, and *Mucispirillum schaedleri* (Figure 8).

### Alpha and beta diversity

**Figure 9:**
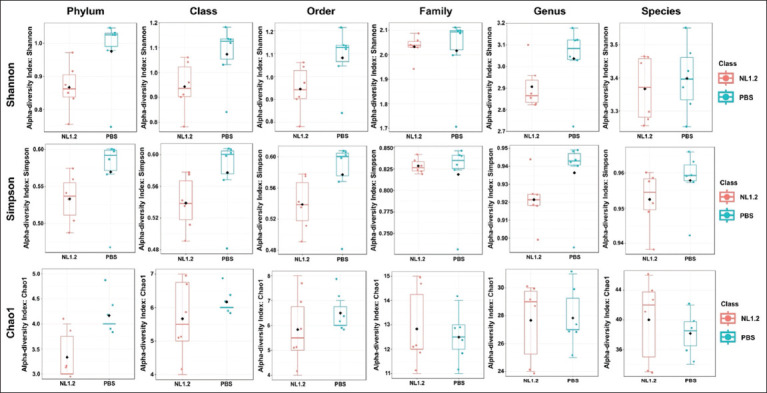
Box plots illustrating the alpha diversity indices (Shannon, Simpson, and Chao1) in the microbiome of mice treated with *Bacillus amyloliquefaciens* NL1.2 compared with the control (phosphate-buffered saline) group. The median is represented by the line in each box, while the whiskers extend to the minimum and maximum values. Outliers and individual sample values are represented as dots.

**Figure 10:**
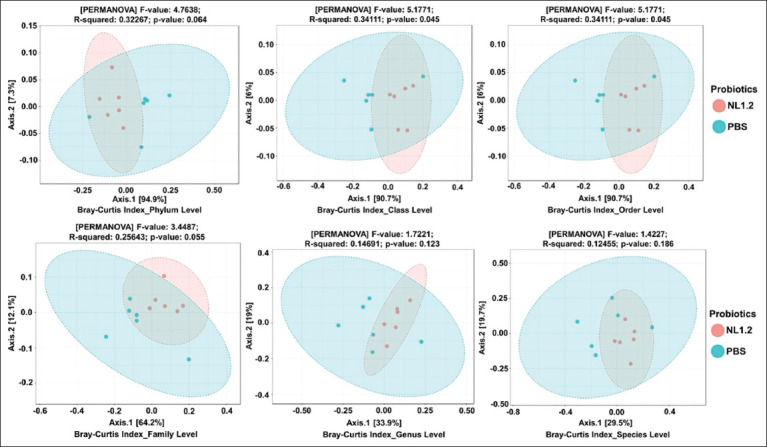
Beta diversity of microbiomes between *Bacillus amyloliquefaciens* NL1.2-treated and control (phosphate-buffered saline) groups, as shown by PCoA plots based on Bray-Curtis index. Ellipses represent confidence intervals, with proximity on the plot indicating similar microbiome compositions.

**Figure 11:**
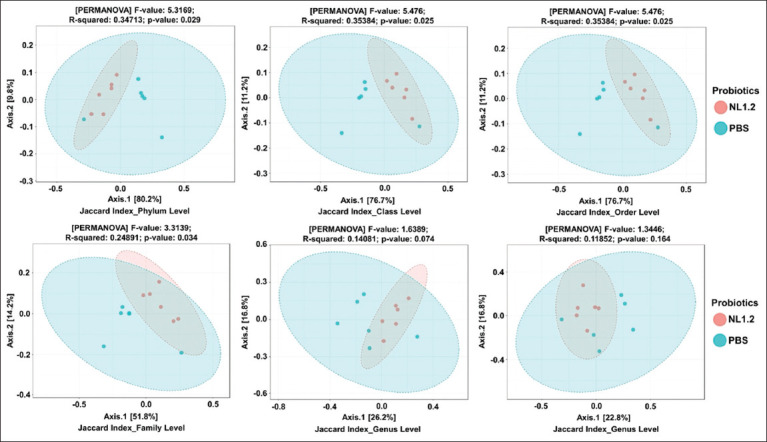
Beta diversity of microbiomes between *Bacillus amyloliquefaciens* NL1.2-treated and control (phosphate-buffered saline) groups, as shown by PCoA plots based on the Jaccard index. Ellipses represent confidence intervals, with proximity indicating similar microbiome compositions.

Alpha diversity (Shannon, Simpson, Chao1 indices) showed no significant differences between groups (p > 0.05) (Figure 9). However, beta diversity analysis (Bray-Curtis and Jaccard) revealed significant compositional differences, particularly from class to family levels, confirming a distinct microbial profile in NL1.2-treated mice (Figures 10 and 11).

## DISCUSSION

### Isolation of spore-forming bacteria from native swine feces

A notable feature of *Bacillus* spores is their intrinsic resistance to wet heat. Sublethal heat treatments, such as exposure to temperatures between 60°C and 75°C for approximately 30 min, can enhance both the rate and extent of germination [44]. In this study, a wet-heat treatment at 65°C for 30 min was applied to eliminate vegetative cells and stimulate spore germination. Heat-treated samples were subsequently cultured aerobically at 37°C to selectively recover *Bacillus* species, while simultaneously excluding obligate anaerobes such as *Clostridium* [45]. A total of 103 isolates were identified as spore-forming *Bacillus* species, a result that aligns with their known thermotolerance and ecological resilience. *Bacillus* probiotics are frequently isolated from the gastrointestinal tracts and feces of various animal species, including pigs, chickens, cows, sheep, turkeys, geese, and camels [46, 47]. In this study, native Thai pigs were selected as the source for probiotic strain isolation due to their distinctive rearing conditions and microbial exposure. Backyard and free-range husbandry practices likely increase native pigs’ exposure to *Bacillus* species from environmental sources such as soil and plant-based feed residues [48]. In addition, native pigs are naturally adapted to hot, humid climates, possess the ability to utilize low-nutrient feed sources, and exhibit inherent resistance to common pathogens and parasites [49]. They are typically raised without antibiotics, thereby reducing the likelihood of selecting for antibiotic-resistant strains. Moreover, native pigs are commonly fed organic farm materials – such as leftovers, fruit and vegetable scraps, rice bran, and other plant biomass – resulting in feces with higher bacterial abundance and diversity compared to pigs raised on commercial farms [50]. These conditions may also promote the presence of *Bacillus* strains capable of producing fiber-degrading enzymes beneficial for animal feed.

### Species identification and exclusion of the *B. cereus* group

Several *Bacillus* species are currently utilized as probiotic dietary supplements in animal feed. However, the potential for some species to produce toxins poses a significant safety concern for consumption. In particular, the *B. cereus* group - which includes *B. cereus*, *B. anthracis*, *B. thuringiensis*, *Bacillus mycoides*, *Bacillus pseudomycoides*, and *B. weihenstephanensis* [51] - is known to produce various enterotoxins and cytotoxins associated with gastrointestinal and systemic diseases in both humans and animals [52]. To identify safe and effective spore-forming *Bacillus* probiotics for animal use, this study initially performed a species-specific PCR assay to exclude strains belonging to the *B. cereus* group. Subsequent species-level identification was confirmed using *16S rRNA* gene sequencing. These steps ensured that only non-pathogenic, spore-forming *Bacillus* strains with probiotic potential were selected for further evaluation.

### Acid and bile tolerance ability

Probiotic bacteria must be able to survive the acidic environment of the stomach to adhere to and colonize the intestinal tract [53]. Although *Bacillus* probiotics in commercial formulations are generally administered in spore form, it is important to assess the viability of vegetative cells under conditions simulating the gastrointestinal environment [9]. In this study, an acid resistance threshold of pH 3.0 was used with a 3-h incubation period to replicate the gastric exposure time [54]. Only three isolates - *B. megaterium* NA9.5, *B. amyloliquefaciens* NL1.2, and *B. subtilis* NM1.5 - showed strong acid resistance, with survival rates exceeding 100%, suggesting that these strains were actively growing under simulated gastric conditions. These results are consistent with previous studies. For example, *Bacillus* species isolated from dairy sludge showed high resistance to simulated gastric juice, maintaining survival rates above 90% after 4 h of incubation. These strains also survived and proliferated in simulated intestinal fluid, achieving survival rates over 145% [55]. Our results showed that *Bacillus* strains exhibited greater acid tolerance than certain commercial probiotic strains as described in a previous study [56]. In addition to acid resistance, bile tolerance is another critical criterion for selecting effective probiotic strains. Bile concentrations in the intestine typically range from approximately 0.2%–2% and play a pivotal role in determining probiotic viability and function [57, 58]. In this study, *B. amyloliquefaciens* NL1.2 and *B. subtilis* NM1.5 demonstrated survival rates exceeding 50% in the presence of bile. This indicates that the vegetative cells of these strains are capable of maintaining membrane integrity in bile salt conditions, facilitating their survival in the intestinal lumen [59], while their spores can germinate effectively in the bile-rich environment of the small intestine [60].

### Adhesion ability

The ability of a probiotic to adhere to the intestinal epithelium is a major factor in selecting strains for application. Adhesion supports transient colonization, modulates immune responses, and enhances gut barrier function and metabolism [61]. Cell surface characteristics such as autoaggregation, hydrophobicity, and biofilm formation are key indicators of probiotic adhesion and are commonly used in *in vitro* screening [53, 62]. In this study, all these properties were evaluated. Autoaggregation is a critical trait that promotes bacterial adhesion to host tissues and excludes enteric pathogens through physical barrier formation [63]. *B. amyloliquefaciens* NL1.2 and *B. subtilis* NM1.5 exhibited time-dependent self-aggregation. Similar observations have been reported for *Bacillus* species isolated from camel milk [64] and carp [65]. Cell surface hydrophobicity is associated with bacterial adhesion, as it facilitates initial contact between the bacteria and intestinal cells. The MATH method is widely used to assess this property [63, 66]. In this study, NL1.2 showed moderate hydrophobicity, while NM1.5 exhibited weak hydrophobicity. This variability reflects differences in the surface composition of the bacterial cell wall, which includes both hydrophilic and hydrophobic elements [67]. Zeng *et al*. [68] have documented hydrophobicity ranges from 14.40% to 81.92% in *Bacillus* from yaks, 33%–74% in strains from Rohu fish [69], and 77%–93% in strains from Idli batter [70]. Factors affecting these values include culture media [62], organic acid content [71], and solvent types [72]. While useful, the MATH assay primarily assesses van der Waals and electrostatic interactions rather than actual hydrophobic bonding, which may limit interpretability [73]. This underscores the importance of using multiple criteria when selecting probiotic strains with strong adhesion characteristics. Whereas hydrophobicity contributes to initial adherence, biofilm formation enhances persistence in the gastrointestinal tract. In this study, both NL1.2 and NM1.5 formed biofilms, with NM1.5 producing significantly more biofilm biomass. Comparable results have been observed in *Bacillus* strains from acidic fermented foods [70]. Although experimental conditions vary, biofilm-forming capacity is advantageous for probiotics as it improves survival, pathogen exclusion, mucosal immunity modulation, and pH tolerance through protective extracellular matrices [74]. Caco-2 cells, which differentiate into monolayers resembling absorptive enterocytes, were used to assess bacterial adhesion *in vitro* [75, 56]. Both *Bacillus* strains exhibited measurable but low adhesion, consistent with literature findings. This suggests the potential for gut colonization and supports their probiotic viability. However, Caco-2 cells lack mucus production, which can influence adhesion levels [75, 76]. by Mingmongkolchai and Panbangred [77] also report low adhesion rates for *Bacillus* spores and vegetative cells from cow milk, pig, and cattle feces (0.23%–3.81%), and strains like *B. indicus* HU36, *B. subtilis* PY79, and *B. subtilis* Natto generally adhere at rates below 1% [78].

### Antimicrobial activity

A crucial characteristic of probiotic strains is their capacity to demonstrate antimicrobial activity against pathogenic microorganisms, which can vary among strains [79]. Probiotics produce various bactericidal or bacteriostatic substances, such as organic acids, bacteriocins, hydrogen peroxide, exopolysaccharides, and other low-molecular-mass compounds, which help control microbial growth and suppress pathogenic bacteria, enhancing microbiological safety [80, 81]. In this study, anti-pathogenic activity was assessed using an agar well diffusion assay. The results showed that CFCS from *B. subtilis* NM1.5 did not inhibit any of the indicator pathogens. In contrast, *B. amyloliquefaciens* NL1.2 exhibited anti-pathogenic activity against all tested pathogens, even after pH neutralization, indicating that the antimicrobial compound is likely not an organic acid. The antimicrobial activity of *B. amyloliquefaciens* NL1.2 is likely attributable to secreted bacteriocins, biosurfactants, or other bioactive metabolites. However, reliance on agar well diffusion assays may not fully represent the activity of compounds with limited diffusibility. Future studies should focus on identifying the specific antimicrobial compounds responsible for this activity, evaluating their stability under different conditions, and employing advanced techniques, such as HPLC or mass spectrometry, to comprehensively characterize these compounds. Lei *et al*. [16] and Golnari *et al*. [47] also reported that *Bacillus* species from different sources, such as dairy products, soil, and animal wastes, exhibit broad-spectrum antimicrobial activity against various pathogenic bacteria, such as *Salmonella* Typhimurium, *Salmonella* Enteritidis, *Klebsiella pneumoniae*, *Enterococcus faecium*, *Staphylococcus aureus*, and *E. coli*. These findings further support the potential of *B. amyloliquefaciens* NL1.2 as a promising candidate for probiotic applications, particularly for controlling pathogenic bacteria.

### Co-aggregation with pathogens

Co-aggregation, a highly specific cell-cell recognition and adhesion process in which genetically distinct bacteria adhere to one another, is another potential mechanism underlying the antagonistic effect against pathogens [82]. This mechanism serves as a protective barrier by preventing pathogen colonization and promoting the persistence of probiotics in the gastrointestinal tract. It is potentially mediated by specific molecules involved in microbial or cellular adhesion, and its effectiveness is strain-dependent [83]. In this study, the co-aggregation abilities of *B. amyloliquefaciens* NL1.2 and *B. subtilis* NM1.5 were evaluated against three pathogens: EHEC SC2451–1, EPEC SC2451–2, and *Salmonella* Typhimurium SC2451–3. Both strains demonstrated moderate co-aggregation percentages, with NL1.2 showing slightly stronger co-aggregation with EHEC SC2451–1 and *Salmonella* Typhimurium SC2451–3, and NM1.5 exhibited higher co-aggregation with EPEC SC2451–2. The observed differences in co-aggregation underscore the strain-dependent specificity of *Bacillus* interactions with enteric pathogens. Previous studies by Pełka *et al*. [84] and Shahbaz *et al*. [85] have emphasized the strain-specific nature of co-aggregation abilities among *Bacillus* species, for example, *B. subtilis*, *B. coagulans*, *B. amyloliquefaciens*, and others have shown varying co-aggregation with pathogens such as *E. coli*, *S. aureus*, *Listeria monocytogenes*, and *Salmonella* species, with differences depending on the strain and incubation time. These findings underscore the critical role of co-aggregation in enhancing the probiotic potential of *Bacillus* strains by preventing pathogen colonization and supporting their establishment within the gastrointestinal tract.

### Safety aspects of *Bacillus* strains

Regarding safety, we evaluated the *Bacillus* strain profiles by assessing hemolytic activity and antibiotic susceptibility. Hemolytic activity, typically assessed on blood agar, is a critical safety parameter, with γ-hemolysis indicating non-pathogenic profiles [86]. Our results showed that both *Bacillus* strains were non-hemolytic, indicating their safety as hosts. This finding is consistent with several previous studies that have reported non-hemolytic *Bacillus* species from various sources, such as *B. subtilis* from Natto [17], *B. velezensis* from poultry feces [72], *B. proteolyticus*, and *B. amyloliquefaciens* from yaks [68]. Although most *Bacillus* species are regarded as safe and widely used as probiotics [87], the possibility of direct horizontal transfer of resistance genes from probiotics to commensal and pathogenic microorganisms in the gut warrants careful consideration in further studies and assessments of probiotic safety [88]. Moreover, evaluating the antimicrobial susceptibility of *Bacillus* strains used in feed additives is strongly recommended [9]. In this study, *B. amyloliquefaciens* NL1.2 was susceptible to all tested antibiotics, indicating its safety. However, *B. subtilis* NM1.5 displayed resistance to erythromycin, a finding consistent with earlier reports. For instance, 51.5% of *Bacillus* species in chicken and pig feces in Vietnam are erythromycin-resistant [89]. Similarly, 18.2% of *Bacillus* species are aquaculture probiotics in China [90]. Erythromycin resistance in *Bacillus* species has been linked to the methylation of 23S rRNA macrolide binding sites mediated by genes such as *ermD* and *ermK* in *B. licheniformis* [91, 92] and the *erm34* gene found in *B. clausii* [93].

### Fiber-degrading enzymes production by *Bacillus* strains

Animal feed traditionally consisted of grains, forage, and silage. However, there is an increasing trend toward using agro-industrial waste and by-products to reduce costs and enhance production [94]. Although these alternative feed sources often have poor nutritional quality and high fiber content, the use of exogenous enzymes has become an essential solution to improve their nutritional value, digestibility, and overall animal performance. These enzymes help digest nutrients, reduce antinutrients, and promote intestinal health in both ruminants and non-ruminants at all stages of growth [2]. Several *Bacillus* species in their vegetative form produce a range of extracellular enzymes, including cellulase, xylanase, amylase, protease, lipase, and phytase, which are crucial for breaking down fiber components such as cellulose, hemicellulose, and pectin in animal feed [95]. In this study, we also assessed the ability of *B. amyloliquefaciens* NL1.2 and *B. subtilis* NM1.5 to produce fiber-degrading enzymes, including cellulase, xylanase, and pectinase. The results illustrated that both strains demonstrated cellulase activity, whereas NM1.5 exhibited higher activities of xylanase and pectinase compared with NL1.2. The capacity of these strains to produce fiber-degrading enzymes may reflect their ecological adaptation to the fiber-rich diets consumed by native pigs. These fiber-degrading enzymes play a significant role in improving nutrition and expanding feed options in livestock systems. In poultry, these enzymes improve digestion and nutrient use by reducing bacterial fermentation, enhancing nutrient absorption, lowering feed viscosity, increasing fiber digestibility, and supporting the gut microbiota [96]. In pigs, exogenous enzymes improve feed digestibility by breaking down complex structures [97], whereas in ruminants, multienzyme complexes improve forage digestibility, starch availability, and overall performance [98]. In addition, these enzymes help with composting by breaking down feed components and reducing the viscosity of raw materials [99, 100]. The ability of both *Bacillus* strains to produce fiber-degrading enzymes highlights their potential to enhance feed quality and digestion across various livestock systems, particularly in industries focused on utilizing cost-effective, hard-to-digest feed materials.

### Safety evaluation of *B. amyloliquefaciens* NL1.2 in mice

*B. amyloliquefaciens* NL1.2, identified as a promising strain through *in vitro* evaluations, was evaluated for safety in a comprehensive 30-day *in vivo* study using a mouse model. The absence of adverse effects on body weight, food consumption, or behavior in probiotic-treated mice highlights the strain’s compatibility and safety. Furthermore, physical examination revealed no abnormalities in appearance, motor activity, or gastrointestinal health, such as diarrhea or other symptoms. Histological analysis corroborated these findings, as no pathological changes were identified in the small intestine, colon, liver, or spleen. Specifically, there were no signs of inflammation, necrosis, or bacterial translocation, highlighting the safety of NL1.2 in maintaining organ integrity and function. These findings align with a previous study by Metlakunta and Soman [101] on *Bacillus* probiotics, such as *B. coagulans* SNZ1969 and *B. subtilis* MB40 [102], which exhibited similar safety profiles and tolerability, supporting the use of *Bacillus* species in probiotic applications.

### Enhancement of intestinal immune response by *B. amyloliquefaciens* NL1.2

In addition to safety, NL1.2 has the potential to enhance the intestinal immune response by increasing secretory IgA production. Secretory IgA is the primary antibody in mucosal secretions produced by intestinal plasma cells, protecting against pathogen adhesion and penetration while regulating the gut microbiota and maintaining homeostasis [103]. The observed increase in sIgA levels in probiotic-treated mice indicates that NL1.2 can strengthen the intestinal immune barrier, potentially improving gut health and resilience to infections. These results align with a prior study by Lai *et al*. [104] on *Bacillus* strains, such as *Bacillus* species DU106 and *B. licheniformis* S6 [105], which have also shown to enhance sIgA production, further supporting the immunomodulatory properties of *Bacillus* probiotics in promoting intestinal homeostasis.

### Effects of *B. amyloliquefaciens* NL1.2 on the gut microbiome

Our study explored the effects of *B. amyloliquefaciens* NL1.2 supplementation on the gut microbiota of mice. After 30 days, microbiome analysis revealed significant alterations in gut microbiota composition, including increased diversity and changes at various taxonomic levels, compared with the control group. Notably, there was an increase in the *Bacteroidetes* phylum, which plays a key role in digesting complex polysaccharides and oligosaccharides. Previous studies have highlighted the importance of *Bacteroidetes* in breaking down fiber-rich foods and producing beneficial nutrients and vitamins for both the host and the microbiota. In addition, *Bacteroidetes* and other anaerobic bacteria help protect against pathogenic microorganisms, supporting long-term gut health [106, 107]. The presence of *B. salanitronis* in the probiotic group further demonstrates the strain’s ability to enhance the breakdown of complex carbohydrates and improve gut health [106]. Similarly, the increased abundance of *B. intestinihominis* emphasizes the role of this bacterium in maintaining gut microbial balance [107]. In contrast, the control group showed an increase in potentially harmful bacteria, such as *Deferribacteres* and *Mucispirillum*, which are associated with inflammatory bowel diseases and infections [108], suggesting that the microbiota structure in the control group may be more prone to disease risk. Although there were no significant changes in alpha diversity between the probiotic and control groups, beta diversity analysis revealed notable differences in the microbiota distribution between the groups. This highlights the effect of probiotics on the overall gut microbiota structure, although there were no changes in overall diversity. From these findings, *B. amyloliquefaciens* NL1.2 has the potential to positively influence the composition of gut microbiota by selectively enriching commensal gut microbes such as *B. intestinihominis* and *B. salanitronis* while reducing potentially pathogenic taxa, supporting its use as a probiotic to enhance gut health and prevent gut-related diseases.

### Implications and future perspectives

The probiotic characteristics exhibited by *B. amyloliquefaciens* NL1.2 underscore its potential as a functional feed additive in livestock production. Its robust tolerance to gastrointestinal stress, capacity to modulate the gut microbiota, promote mucosal immunity, and adhere to intestinal epithelial cells, combined with a favorable safety profile - especially sensitivity to commonly used antibiotics - make it a compelling candidate in the shift toward more sustainable animal health strategies. As the livestock industry moves away from routine antibiotic use, strains like NL1.2 offer a viable alternative for enhancing animal health and productivity.

Future research should focus on optimizing the formulation and delivery of NL1.2 in animal feed and assessing its long-term impacts on animal performance, disease resistance, and overall health under practical farming conditions. Moreover, exploring the synergistic effects of co-administration with other probiotics or functional feed ingredients may further improve its efficacy. In response to increasing consumer demand for antibiotic-free and environmentally responsible livestock production, NL1.2 represents a promising tool in the development of next-generation feed solutions aligned with global sustainability goals.

## CONCLUSION

This study identified and characterized *B. amyloliquefaciens* NL1.2, a spore-forming bacterium isolated from the feces of native Thai pigs, as a promising probiotic candidate for use in livestock. The strain demonstrated excellent tolerance to acidic (pH 3.0) and bile (1%) conditions, with survival rates exceeding 100%–75%, respectively, indicating its potential to endure gastrointestinal transit. NL1.2 exhibited strong autoaggregation (65.99%), moderate cell surface hydrophobicity (34.13%), biofilm-forming capacity (OD_570_ = 0.76), and measurable adhesion to Caco-2 cells (2.0%), all of which suggest a capacity for intestinal colonization. Furthermore, it displayed broad-spectrum antimicrobial activity against key enteric pathogens (EHEC, EPEC, and *Salmonella* Typhimurium), alongside moderate co-aggregation ability.

NL1.2 was non-hemolytic (γ-hemolysis) and susceptible to all tested antibiotics, fulfilling key safety prerequisites. It also produced fiber-degrading enzymes, including cellulase, xylanase, and pectinase, supporting its utility as a functional feed additive. *In vivo* administration to mice over 30 days revealed no adverse clinical, behavioral, or histological effects, confirming its systemic safety. Notably, NL1.2 significantly increased intestinal secretory IgA levels (18.70 ng/mL vs. 11.76 ng/mL in controls; p < 0.05), highlighting its potential immunomodulatory effects. Gut microbiome analysis further revealed a favorable modulation in microbial composition, characterized by the enrichment of beneficial taxa (*B. salanitronis* and *B. intestinihominis*) and suppression of potentially pathogenic groups (*Deferribacteres* and *M. schaedleri*), with significant differences confirmed through LEfSe and beta diversity analyses.

Strengths of this study include the integration of rigorous *in vitro* and *in vivo* evaluations, taxonomically resolved gut microbiome profiling, and comprehensive functional characterization of probiotic traits relevant to livestock application. However, the limitations include the use of a murine model rather than the target host species, lack of quantification of specific antimicrobial compounds, and absence of performance-related metrics (e.g., growth and feed conversion) in animals.

*B. amyloliquefaciens* NL1.2 satisfies key probiotic criteria, demonstrating strong functional potential, safety, and microbiota-modulating capacity. Its properties make it a viable candidate for development as a feed additive in antibiotic-free livestock production. Future studies should focus on host-specific trials in target species, scale-up fermentation, formulation optimization, and long-term efficacy under field conditions to realize its full application potential.

## DATA AVAILABILITY

All the generated data are included in the manuscript.

## AUTHORS’ CONTRIBUTIONS

KK and ML: Conception and design of the study. KK and ML: Conducted the study and analyzed and interpreted the data for the *in vitro* assessment. KK, ML, and MT: Conducted the *in vivo* assessment and interpreted the results. KK, ML, SP, and VS: Conducted the gut microbiome analysis and interpreted the data. KK: Drafted the manuscript. and ML, MT, and SP: Critically reviewed and revised the manuscript. All authors have read and approved the final manuscript.
